# A Retrospective Analysis on Cervical Spine Magnetic Resonance Imaging Findings in Patients with Neck Pain in a Tertiary Hospital, Addis Ababa, Ethiopia

**DOI:** 10.4314/ejhs.v31i5.15

**Published:** 2021-09

**Authors:** Tewodros Endale Balcha, Ferehiwot Bekele Getaneh, Abebe Mekonnen Woldeyohannes

**Affiliations:** 1 Department of Radiology, College of Health Sciences, Addis Ababa University, Addis Ababa, Ethiopia

**Keywords:** Cervical spine, Magnetic resonance imaging, Neck pain

## Abstract

**Background:**

Neck pain is a common health problem throughout the world causing significant individual disability and economic burden on health care facility. Many factors are mentioned as a cause or association in relation to neck pain, of which degenerative and posttraumatic cause are the main ones. The aim of this study is to assess cervical spine Magnetic Resonance Imaging (MRI) patterns in patients presented with neck pain.

**Methods:**

A retrospective analysis of 160 patients who had cervical spine Magnetic Resonance Imaging (MRI) for evaluation of a neck pain was done. The study was conducted between February to August 2018 at Tikur Anbessa Specialized Hospital. The patients' clinical history and magnetic resonance imaging reports were reviewed from their medical records. All patients who have cervical spine Magnetic Resonance Imaging (MRI) for a neck pain were included in the study. Those patients with acute traumatic neck pain were excluded.

**Results:**

From a total of 160 patients, 71(44.4%) were males and 89(55.6%) were females. Degenerative cervical spine findings such as intervertebral disc degenerations were seen in 127(79.4%) patients. Non-degenerative imaging findings such as neoplasm and infection were seen in 10(6.3%) patients only. The MRI was normal in 23(14.4%) of them.

**Conclusion:**

The most common cause of neck pain from this study is degenerative changes of the cervical spine, which was predominant in the older age groups. Non-degenerative causes such as neoplasm and infection were less common findings.

## Introduction

Neck pain is a common health problem throughout the world causing significant individual disability as well as economic burden on health care facility. In the Global Burden of Disease 2010 Study, neck pain is the 4^th^ highest in terms of disability as measured by YLDs (Years Lived with Disability), and 21st interms of overall burden (1–4).

There is considerable variability in the epidemiologic studies of neck pain in the world literatures. An overall prevalence of 0.4% to 86.8% and annual prevalence of 30%–50 % to 4.8 % to 79.5 % is mentioned in the general population. Women and middle age individuals are more commonly affected (2, 5–8).

There are many factors listed as a cause or associated factors in relation to neck pain. Post traumatic and degenerative causes are two major areas of perspectives mentioned as etiologic entities. Degenerative changes in patients with chronic neck pain include degenerative disc disease and disc herniation. Degenerative changes can also be caused by prior injury (9, 10).

Considering the magnificent soft tissue resolution of magnetic resonance imaging (MRI); it is said to be a very useful imaging modality in the evaluation of patients with neck pain to assess abnormalities such as disk herniations, canal encroachment by osteophytes, tumor or infections and ligament abnormalities (9).

As per the knowledge of the authors to date; there is no single study dedicated to MRI patterns of cervical spine in patients with neck pain in Ethiopia despite its impact on health care and economic burden stated worldwide. Our study evaluated the common cervical spine MRI abnormalities seen in patients with neck pain.

## Methods and Materials

The study was conducted at Department of Radiology, Tikur Anbessa Specialized Hospital (TASH), Addis Ababa, Ethiopia from February to August 2018. The hospital is the largest tertiary level referral and teaching hospital in the country. It is one of the centers of excellence in Ethiopia in undergraduate, post graduate and subspecialty programs in health sciences. The radiology department is one of the many departments in the institution with experienced radiologists. The department is equipped with high-tech radiologic machines including MDCT (Multi Detector Computed Tomography) machines with 64 and 128 slices and high-resolution MR scanner with 1.5 tesla strength.

Retrospective descriptive study was conducted by reviewing cervical spine MRI reports of patients with neck pain presented to department of radiology. Cervical spine MR images were acquired using Philips Medical Systems Achieva 16 channels 1.5 T strength MR scanner with standard neck and brain receiving coils. All patients, who had cervical spine MRI evaluation for an indication of neck pain during the period under review, were included in this study. Patients with acute trauma to the neck and those with primary complaint other than neck pain were excluded from the study. The cervical spine MRI images were interpreted by neuroradiologists with more than 10 years of experience and fellows in neuroradiology. MRI reports and clinical data records of patients with neck pain were retrieved from the radiology department patient record chart and recorded on a well-prepared format designed to include patients' sex, age, clinical indication and imaging findings by the principal investigator.

**Data processing and analysis;** The data was cleaned, coded and entered to statistical package for social sciences (SPSS) version 20 for analysis. Frequencies, mean, standard deviation, percentages and cross tabulation were determined and summarized. Association studies between some of the variables were done using chi square test using P-value of <0.05 as statistically significant association.

**Ethical considerations**: Data collection was started after getting permission from the ethical review committee of the department of radiology at Addis Ababa University. Patient confidentiality was maintained by omitting patients' name and hospital identification number from data collecting format and selected representative images.

## Results

From a total of 160 patients in this study 71(44.4%) were males and 89(55.6%) were female patients. The mean age was 46.3 ± 13.3years with a range of 18–85 years. Participants in age range of 41–50 years were 31.9%, whereas younger ages (<21 years) were 1.9%. A 2.5% of the participants were >70 years of ages.

Degenerative cervical spine findings with resultant neural foramina and/or spinal canal stenoses were seen in 127(79.4%) patients, among these, 68(53.5%) were female and 59(46.5%) were male. Non-degenerative imaging abnormalities such as neoplasm and infection were seen in 10(6.3%) patients. Cervical spine MRI was normal in 23(14.4%) of them.

**Figure 1:**
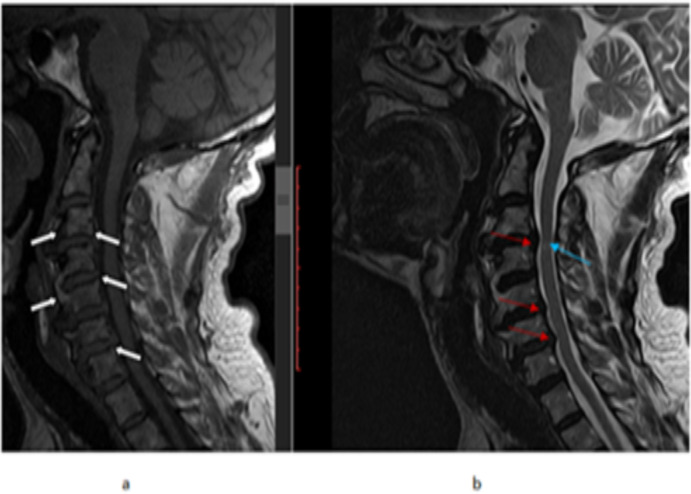
A 55 year old male patient, cervical spine MRI (a) T1W sagittal image showing multiple level anterior and posterior marginal osteophytes (white arrows) (b) T2W sagittal image showing decreased intervertebral disc signal and height from C2-C3 to C6-C7 levels with posterior disc osteophyte complexes at C3-C4, C5-C6 and C6-C7 levels(red arrows). There is also focal spinal cord signal change and decreased caliber at C3-C4 level (blue arrow).

Intervertebral disc (IVD) degenerative changes in the form of desiccation/dehydration, IVD loss of height and disc osteophyte complex in combination or alone were seen in 126(78.8%) patients (Table 1, Figure 1) and all are multilevel, involving more than one intervertebral disc level. Vertebral bone degeneration in the form of posterior marginal osteophytes alone or in combination with vertebral body loss of height was seen in 120(75%) patients.

**Table 1:** Frequency of imaging findings of Intervertebral disc (IVD) degeneration of 160 patients who came for cervical MRI evaluation at TASH 2017–18

IVD degenerative finding	Frequency	Percentage
IVD desiccation	87	54.4%
IVD space loss of height	45	28.1%
Disc-osteophyte complex	109	68.1%
Disc osteophyte complex with disc dehydration and/or loss of height	75	46.9%

A disc osteophyte complex with either disc dehydration and/or loss of height was seen in 75(46.9%) patients, with age group > 40 years contributing 85.3%. There was no statistically significant difference among female and male participants regarding IVD degenerative changes (P=0.112).

**Figure 2:**
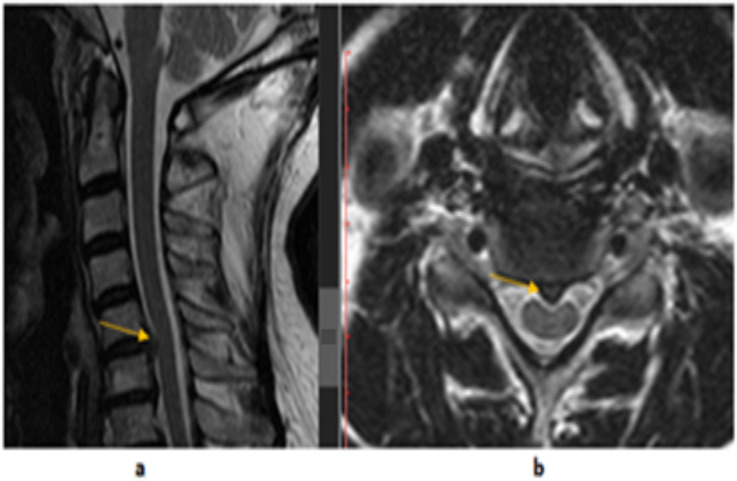
A 52-year-old female patient, (a) T2W sagittal and (b) T2W axial, cervical spine MRI images. A C5-C6 level posterior central disc protrusion (arrows), which cause spinal canal stenosis and cord compression. A decreased signal of the intervertebral discs and straightening of cervical spine alignment are demonstrated

A disc osteophyte complex with either disc dehydration and/or loss of height was associated with age group of > 40 years (P< 0.0001). Intervertebral disc herniations excluding disc osteophyte complexes were seen in 18(11.25%) patients, out of which 8(44.4%) were male and 10(55.6%) were female. A 38.9% of them were observed in the 5^th^ decade followed by the 3^rd^ decade (27.8%). Only 1(5.6%) patient was seen in age <21 years and no patient seen in age >60 years (Table 2). C5-C6 level involved in 50 % of them, followed by C4-C5 (44.4%) and C6-C7 (22.2%) levels (Table 3, Figure 2).

**Table 2:** Distribution of Intervertebral disc (IVD) herniation according to age group in 160 patients with neck pain who came for cervical spine MRI evaluation at TASH in 2017–18

Age group	Frequency	Percent
<21	1	5.6
21–30	5	27.8
31–40	2	11.1
41–50	7	38.9
51–60	3	16.7
Total	18	100.0

**Table 3:** Distribution of Intervertebral disc (IVD) herniation according to disc level in 160 patients with neck pain who came for cervical spine MRI evaluation at TASH in 2017–18

Disc level	Frequency	Percent of Cases
C2-C3 LEVEL	2	11.1%
C3-C4 LEVEL	5	27.8%
C4-C5 LEVEL	8	44.4%
C5-C6 LEVEL	9	50.0%
C6-C7 LEVEL	4	22.2%

Single level herniations were seen in 11(61.1%) as compared to multiple level herniations which were seen in 7(38.9%) patients. Neural foramina and spinal canal stenoses were seen in 117(73.1%) and 110(68.8%) patients respectively. Imaging findings suggesting spinal cord compression with signal change were seen in 24(15%) patients. Spondylolisthesis was seen in 2(1.3%) patients. Findings suggesting spine tuberculous infection were seen in 3(1.9%) patients. Neoplasms were suspected in 3(1.9%) patients. Other non-degenerative abnormalities categorized under miscellaneous were seen in 4(2.5%) patients. There were three cases of Chiari I malformation (two with syrinx) and one case of congenital block vertebra.

## Discussion

The role of cervical spine MRI for evaluation of neck pain has been described in many literatures considering its excellent image contrast in demonstrating IVD, spinal cord, vertebral bone marrow, foraminal stenosis and other many aspects of anatomies such as ligamentous structures (9, 11, and 12). This study assessed cervical spine MRI examinations done for patients presented with neck pain and the result showed degenerative spine disease is the most common cause of neck pain, which is in consistent with previous teaching hospital based retrospective and prospective studies done in Nigeria and Dhaka respectively (13–15).

Intervertebral disc degenerative findings in the form of desiccation/dehydration, loss of height and disc osteophyte complex in combination or alone represent the most common degenerative findings (78.8%) with involvement of more than one-disc level in all cases. Increased prevalence of degenerative findings was seen with age, especially with age above 40 years. This is a similar finding with previous retrospective studies done in Nepal and Nigeria on patients with neck pain that undergo cervical spine MRI evaluation showing degenerative changes such as intervertebral disc desiccation and bulge to have increased prevalence with age (10,13).

IVD herniation was seen in 18(11.2%) patients with the highest number of patients seen in the 5^th^ decade (38.9%) followed by the 3^rd^ decade (27.8%). The C5-C6 level was the most commonly affected level (56.5%) and single level involvement was more common than multiple level involvements. Similar epidemiologic finding was seen in a previous study that included 750 symptomatic patients and showed 159 patients to have disc herniations and C5-C6 level as the most common involved level. Multiple level involvement was seen only in 23 patients (10). Disc herniation was found to be exclusively found in symptomatic patients versus asymptomatic patients in a comparative study while other degenerative disc changes were equally prevalent among both symptomatic and asymptomatic patients (16).

Non-degenerative abnormalities were not common findings. They were seen in 10(6.3%) patients. Two studies on patients with neck pain found non-degenerative pathologies in 12.1% and 35.5% respectively (14, 17). The relative less percentage of non-degenerative pathologies in our study may be explained by the exclusion of patients with neck trauma, as majority of patients with non-degenerative findings seen in the above-mentioned studies represent trauma related findings.

MRI is said to be the modality of choice for imaging spine infections with several imaging findings, such as disc space sparing, multilevel subligamentous spread, and large abscesses, can suggest the diagnosis of tuberculous over pyogenic infection, the two most common spine infections (18). In this study imaging findings suggesting infections were detected in 3(1.9%) patients which is a similar prevalence to a previous study (17).

Imaging findings suggesting neoplasm were seen in three (1.9%) patients, two intramedullary neoplasm suggesting glioma and one vertebral metastasis with similar prevalence to previous studies (14, 15).

Out of the 160 patients in this study, 23(14.4%) patients had no remarkable cervical spine MRI findings. The absence of abnormal imaging findings in these patients may be related to different nonstructural factors such as patient background situations including psychosocial, demographic and socioeconomic factors as many previous studies tried to link up and explain neck pain with different perspectives (2, 19).

This study has a number of limitations. This study didn't also follow the treatment given to see patient response, especially those patients with degenerative findings. Control group would have been a good input to compare patterns of degenerative spine changes, especially in relation to advancing age. Detailed clinical backgrounds of patients such as socioeconomic and psychosocial factors were not considered in this study which would have been useful to assess these factors in relation to the pattern of imaging findings among study individuals.

In conclusion degenerative cervical spine findings were the most common MRI patterns seen in symptomatic patients in this study showing increasing prevalence with age. Nondegenerative imaging findings such as neoplasm and infection were less common findings.

## References

[1] Goode AP, Freburger J, Carey T (2010). Prevalence, practice patterns, and evidence for chronic neck pain. Arthritis Care & Research.

[2] Hogg-Johnson Sheilah, van der Velde Gabrielle, Carroll Linda J, Holm Lena W, David Cassidy J, Guzman Jamie (2009). The Burden and Determinants of Neck Pain in the General Population: Results of the Bone and Joint Decade 2000–2010 Task Force on Neck Pain and Its Associated Disorders. Journal of Manipulative and Physiological Therapeutics.

[3] Abdulkarim JA, Dhingsa R, Finlay DB L (2003). Magnetic Resonance Imaging of the Cervical Spine: Frequency of Degenerative Changes in the Intervertebral Disc with Relation to Age. Clinical Radiology.

[4] Damian Hoy, March Lyn, Woolf Anthony, Blyth Fiona, Brooks Peter, Smith Emma (2014). The global burden of neck pain: estimates from the global burden of disease 2010 study. Ann Rheum Dis.

[5] Hoy D.G, Protani M, De R, Buchbinder R (2010). The epidemiology of neck pain. Best Practice & Research Clinical Rheumatology.

[6] Fejer R, Kyvik KO, Hartvigsen J (2006). The prevalence of neck pain in the world population: a systematic critical review of the literature. European Spine Journal.

[7] El-Sayed Abdulrahman M, Hadley Craig, Tessema Fasil, Tegegn Ayalew, Cowan John A, Galea Sandro (2010). Back and neck pain and psychopathology in rural sub-Saharan Africa: evidence from the Gilgel Gibe Growth and Development Study, Ethiopia. Spine.

[8] Tafese Ararso, Nega Anisha, Kifle Manay, Kebede Wakjira (2014). Predictors of occupational exposure to neck and shoulder musculoskeletal disorders among sewing machine operators of garment industries in Ethiopia. Science Journal of Public Health.

[9] Daffner RH (2010). Radiologic evaluation of chronic neck pain. Am Fam Physician.

[10] Karki D B, Gurung G, Adhikary K P, Ghimire R K (2016). Magnetic Resonance Imaging Findings in Degenerative Disc Disease of Cervical Spine in Symptomatic Patients. Journal of Nepal Health Research Council.

[11] McDonald Marin A, Kirsch Claudia F E, Amin Beejal Y, Aulino Joseph M, Bell Angela M, Cassidy R Carter (2019). ACR Appropriateness Criteria® cervical neck pain or cervical radiculopathy. Journal of the American College of Radiology.

[12] Laker SR, Concannon LG (2011). Radiologic evaluation of the neck: a review of radiography, ultrasonography, computed tomography, magnetic resonance imaging, and other imaging modalities for neck pain. Physical Medicine and Rehabilitation Clinics.

[13] Mustapha Z, Okedayo M, Ibrahim K, Abba Ali A, Ahmadu MS, Abubakar A (2014). Cervical spine MRI findings in patients presenting with neck pain and radiculopathy. Int Res J Basic Clin Stud.

[14] Olarinoye-Akorede S, Ibrahim M, Kajogbola G (2018). Cervical Spine MRI findings in the evaluation of persistent neck pain in a Nigerian Tertiary Hospital. Nigerian Journal of Basic and Clinical Sciences.

[15] Islam MK, Alam SZ, Rahman MS, Akter A (2009). MRI evaluation of neck pain. Journal of Armed Forces Medical College, Bangladesh.

[16] Siivola Sari M, Levoska Sinikka, Tervonen Osmo, Ilkko Eero, Vanharanta Heikki, Keinänen-Kiukaanniemi Sirkka (2002). MRI changes of cervical spine in asymptomatic and symptomatic young adults. European spine journal.

[17] Karki DB, Panta OB, Gurung G (2015). Non degenerative disease in MRI cervical spine of symptomatic patients. Journal of College of Medical Sciences-Nepal.

[18] Diehn FE (2012). Imaging of spine infection. Radiologic Clinics.

[19] Genebra Caio Vitor Dos Santos, Maciel Nicoly Machado, Bento Thiago Paulo Frascareli, Simeão Sandra Fiorelli Almeida Penteado, De Vittaa Alberto (2017). Prevalence and factors associated with neck pain: a population-based study. Brazilian journal of physical therapy.

